# Four new species of Aspidiotini (Hemiptera, Diaspididae, Aspidiotinae) from Panama, with a key to Panamanian species

**DOI:** 10.3897/zookeys.1047.68409

**Published:** 2021-06-24

**Authors:** Jiufeng Wei, Scott A. Schneider, Roxanna D. Normark, Benjamin B. Normark

**Affiliations:** 1 College of Plant Protection, Shanxi Agricultural University, Taigu, Shanxi, 030801, China; 2 USDA, Agricultural Research Service, Henry A. Wallace Beltsville Agricultural Research Center, Systematic Entomology Laboratory, Building 005 – Room 004, 10300 Baltimore Avenue, Beltsville, MD, 20705, USA; 3 Department of Biology, University of Massachusetts, Amherst, MA 01003, USA; 4 Graduate Program in Organismic and Evolutionary Biology, University of Massachusetts, Amherst, MA 01003, USA

**Keywords:** Armored scale insect, biogeography, Coccoidea, Coccomorpha, Neotropics, taxonomy

## Abstract

Four new species of armored scale insect, *Clavaspisselvatica***sp. nov.**, *Clavaspisvirolae***sp. nov.**, *Davidsonaspistovomitae***sp. nov.**, and *Rungaspisneotropicalis***sp. nov.**, are described and illustrated from Panama. We also transfer two previously described species of Panamanian Aspidiotini to new genera, *Hemiberlesiacrescentiae* (Ferris) **comb. nov.** and *Rungaspisrigida* (Ferris) **comb. nov.**, and report the first record of *Selenaspidopsisbrowni* Nakahara in Panama. A key to the species of Aspidiotini occurring in Panama is provided.

## Introduction

Armored scales are the most species-rich family of scale insects, comprising over 2600 species in 418 genera (García Morales et al. 2016). The family is characterized by the complete loss of legs and reduction of antennae in adult females, fusion of the posterior abdominal segments into a pygidium, and the formation of a waxy test (Takagi 1990). Like all members of the suborder Sternorrhyncha, armored scales are strictly phytophagous. Many species of armored scales are pests of agricultural commodities (Miller and Davidson 2005). Heavy infestations inhibit photosynthesis through chlorophyll depletion and crowding of leaf surfaces, reducing plant vigor. Additionally, visible infestations and damage reduce the value of produce and nursery stock (Kosztarab 1990; Miller and Davidson 2005). However, unlike most scale insects, armored scales do not contribute to the growth of sooty molds on hosts because they do not produce honeydew (Henderson 2011).

Some armored scale insect species are extremely polyphagous, with host ranges among the widest known for any herbivorous insect, comprising in some cases over 100 families of plants (Normark and Johnson 2011; Ross et al. 2013; García Morales et al. 2016). The most highly polyphagous species have a strong tendency to be economic pests (Normark and Johnson 2011; Ross et al. 2013; Normark et al. 2014). Because armored scale insects appear to have essentially random dispersal via windblown larvae, Hardy et al. (2015) hypothesized that their host ranges are likely to reflect the plant diversity of their habitats, and that extreme polyphagy may have evolved in habitats with extreme plant diversity, such as tropical rainforests. Because of the economic importance of armored scale insects, they have been extensively sampled on cultivated plants, especially orchard crops and ornamentals (Rosen 1990; Miller and Davidson 2005). But their diversity, abundance, and host associations in natural environments are poorly known, and this is particularly true for tropical rainforests. Since 2010, one of us (BBN) has been systematically sampling armored scale insects in tropical forests. Two of the goals of this effort are to test for cryptic diversity within apparently polyphagous species and to test whether a species’ local abundance is correlated with its host range. Results of tests of these hypotheses using samples from Panama and Borneo are reported in Peterson et al. (2020). Briefly, cryptic diversity is found within some apparently polyphagous species within their native ranges, but some invasive species are truly polyphagous. And local abundance is positively correlated with host range. Another goal of the rainforest sampling effort is to discover and describe new species of armored scale insects, which is the purpose of this article. Specifically, here we describe four new species within the tribe Aspidiotini collected from Panama.

Armored scales are currently classified into four subfamilies: Ancepaspidinae, Aspidiotinae, Diaspidinae, and Furcaspidinae (Normark et al. 2019). Aspidiotini is a large tribe within subfamily Aspidiotinae that includes many pest species that are globally invasive and economically damaging (Schneider et al. 2018). To date, 54 species of Aspidiotini in 16 genera have been recorded from Panama (García Morales et al. 2016; last accessed 31.iii.2021). In addition to the descriptions of four new species, this article includes the first report of *Selenaspidopsisbrowni* Nakahara from Panama. Additionally, this article assigns two Neotropical species to the genus *Rungaspis*, whose species are otherwise restricted to Africa and the southwestern Palearctic. With these records included, 58 species from 18 genera in Aspidiotini are known to occur in Panama, comprising roughly half of the total armored scale fauna for this country (58 out of 118 species reported in ScaleNet) (García Morales et al. 2016). The majority of these species are likely native to the Neotropics (Ferris 1941, 1942; Deitz and Davidson 1986), but many are broadly distributed and are considered major, minor, or potential pests (Miller and Davidson 1990; Schneider et al. 2019). Species that are non-native to this region include members of *Aspidiella*, *Aspidiotus*, *Chrysomphalus*, and *Selenaspidus*, which are widespread pests likely originating from the Australasian, Oriental, and Afrotropical regions (Schneider et al. 2018). An identification key to the species of Aspidiotini found in Panama is provided.

## Material and methods

The sampling locality for new species described in this paper was the canopy crane in San Lorenzo National Park, Colón (9.2802°N, 79.9754°W). The locality was chosen because it offered access to the canopy via the crane and because every tree was reliably identified to species. The first survey was conducted in June 2012 by Geoffrey E. Morse and BBN, and the second in January 2015 by G. E. Morse, Daniel A. Peterson, Hannah Shapiro, and Shannon Trujillo. A full description of the sampling protocol is given in Peterson et al. (2020). Briefly, in each survey, investigators sampled all the tree species accessible from the canopy crane, and sampled multiple individuals of the more abundant species. Foliage of each sampled tree was searched visually for 20 person-minutes. Leaves that appeared to be infested with armored scale insects were collected into plastic bags, along with a 20 cm twig sample and 20 cm^2^ bark sample. Collected material was refrigerated and examined under a dissecting microscope within 5 days; live armored scale insects were transferred to 100% ethanol. Subsequently, sampled scale insects were subjected to a joint morphological / molecular sample preparation that resulted in a sample of purified genomic DNA and a permanent microscope slide mount of the specimen’s cuticle, following the method described in Normark et al. (2019).

In this paper, morphological terminology conforms to descriptions and illustrations provided by Schneider et al. (2019) and Miller and Davidson (2005). Vouchering of specimens was completed following the protocols described by Normark et al. (2019). Measurements were made on a Zeiss Axio Imager.M2 (Carl Zeiss Microscopy, LLC, White Plains, NY, USA) microscope with the aid of an AxioCam and AxioVision software. Illustrations were made using a Nikon Optiphot compound microscope (Nikon USA, Melville, NY, USA) with the aid of a camera lucida. Slide-mounted specimens were examined by the authors under phase contrast and DIC microscopy. The abbreviations L1, L2 and L3 refer to the median, second, and third pygidial lobes, respectively.

Depositories are abbreviated as follows:

**MIUP** Museo de Invertebrados G. B. Fairchild, Panama City, Panama;

**UMEC**University of Massachusetts Entomology Collection, Amherst, Massachusetts, USA;

**USNM**United States National Museum, scale insect collection at USDA Agricultural Research Service, Beltsville, Maryland, USA.

## Taxonomy

### Clavaspis selvatica

Taxon classificationAnimaliaHemipteraDiaspididae

Wei, Schneider, Normark & Normark sp. nov.

AFF1B59A-E64D-5FE5-BFC4-2D6D39A90920

http://zoobank.org/CDB99B24-3013-45F7-AA42-FE31CA298219

Figure 1

#### Material examined.

***Holotype*:** Panama • 1 adult female; Parque Nacional San Lorenzo Canopy Crane, Colón; 9.2802°N, 79.9754°W; 15.i.2015; DA Peterson, GE Morse, H Shapiro, S Trujillo leg.; on *Embothriumcoccineum*; MIUP (D6581C). ***Paratypes***: • 1 adult female with second-instar exuviae; same data as holotype; USNM (D6581A); • 2 adult females; same data as holotype; UMEC (D6581B, D6581E).

#### Description

**(*N* = 4). Adult female** not pupillarial. Appearance in life not recorded. Slide-mounted adult female 670–1450 μm long (holotype 670), 560–1100 μm wide (holotype 560), broadest at mesothorax or metathorax. Body outline turbinate to nearly oval. Derm membranous throughout at maturity except for pygidium. Antennae simple, each with one long seta. Distance between antennae 100–180 μm. Without disc pores associated with anterior or posterior spiracles. ***Lobes***. L1 well developed, slightly wider than long, inner margins near parallel, with 1 notch on each side or without notches, rounded apically; space between lobes approximately 0.25 times width of L1. L2 and L3 absent. ***Plates*** cylindrical, narrow, pointed at apex, simple or with a few fine tines, about as long as L1; 2 plates present in first space, often with 1 or 2 tines near apex giving bifurcate or trifurcate appearance; 1 or 2 plates present in second space, simple or with minute tines; plates absent between L1. ***Ducts*.** Dorsal macroducts of 1-barred type, with 2–3 macroducts arising from first space, 8–10 arising from second space, and 7–8 arising from third space in singular rows. Series of marginal macroducts with wide orifices extending from mesothorax to abdominal segment II; at least two present per segment. Groups of ventral submarginal microducts occurring on head, thorax, and abdominal segments I–V. ***Paraphyses*.** With 1 pair of paraphysis-like basal scleroses near mesal margins of L1; 1 pair of paraphyses in first space, paraphysis arising from lateral margin of L1 slightly longer than paraphysis arising from medial margin of L2, both mushroom-like in shape with distinctive dome or cap at anterior end; 1 pair of small clavate paraphyses in second space. ***Anal opening*** longer than wide, 11–14 μm long, 5–7 μm wide, positioned 17–25 μm (1.5–2 anal lengths) from the base of L1, located within posterior third of pygidium. ***Perivulvar pores*** few, 2–6 pores in total, divided into 2–4 groups, with 1–4 in each group.

#### Remarks.

This new species is most similar in appearance to *C.coursetiae* (Marlatt) with subtle differences distinguishing the two. Submarginal groups of microducts form a semicircle around the head, thorax, and pre-pygidial abdominal segments of *C.selvatica* but are more diffusely scattered in *C.coursetiae*, not organized in an obvious semicircular ring. In *C.selvatica*, at least two large macroducts are present on the mesothorax, while in *C.coursetiae* only one at most is present, falling near the posterior margin of the mesothorax. The plates are nearly as long as L1 and fringed in *C.selvatica* but are short and simple in *C.coursetiae*. This species is also similar to *C.subsimilis* (Cockerell) in body shape and the shape of L1 but can be distinguished by possessing perivulvar pores on the pygidium (absent in *C.subsimilis*).

#### Host plant.

*Apeibaaspera* Aubl. (family Malvaceae).

#### Etymology.

The epithet *selvatica* is the Latin adjective meaning wild, literally “of the forest” (*selva*). Our choice of this name is influenced by the fact that in modern Spanish, the word *selva* is identical to its Latin ancestor in form, but now refers specifically to tropical rainforest.

#### Distribution.

Panama (Colón).

**Figure 1.**
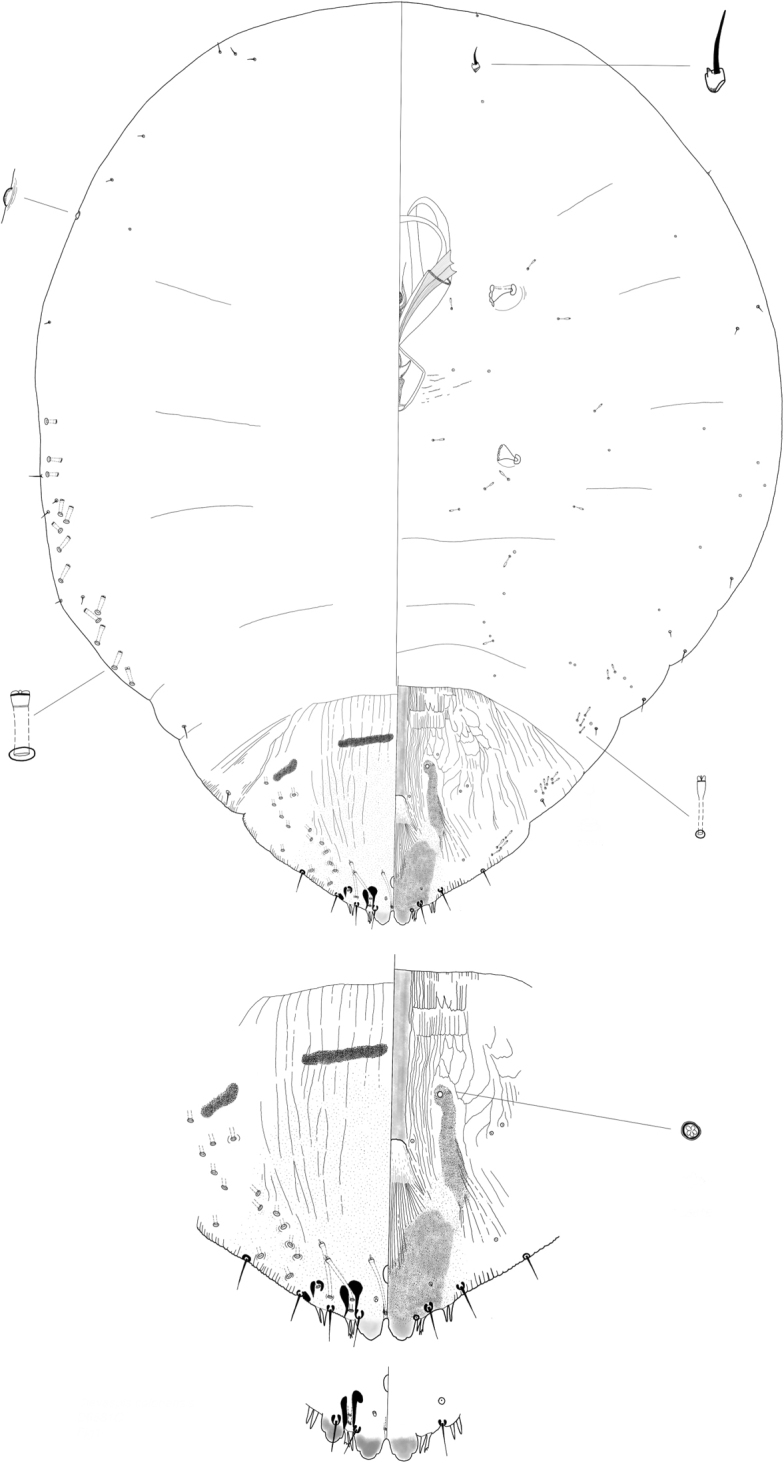
*Clavaspisselvatica* Wei, Schneider, Normark & Normark sp. nov. Adult female, full body view, illustrated from the holotype (D6581C); expanded views of pygidium showing variation, illustrated from the holotype (D6581C) and a paratype (D6581E).

### Clavaspis virolae

Taxon classificationAnimaliaHemipteraDiaspididae

Wei, Schneider, Normark & Normark sp. nov.

6755DF26-1264-5232-95C2-BAF943BE77AC

http://zoobank.org/EEB45109-341D-44C4-98B8-E1BEC59F0CB1

Figure 2

#### Material examined.

***Holotype*:** Panama • 1 adult female; Parque Nacional San Lorenzo Canopy Crane, Colón; 9.2802°N, 79.9754°W; 17.i.2015; DA Peterson, GE Morse, H Shapiro, S Trujillo leg.; on *Virolamultiflora*; MIUP (D6676B). ***Paratype***: • 3 adult females; same data as holotype; USNM (D6676A, D6676D, D6677A); • 3 adult females; same data as holotype; UMEC (D6674G, D6676C, D6677C).

#### Description

**(*N* = 7). Adult female** not pupillarial. Appearance in life not recorded. Slide-mounted adult female 475–900 μm long (holotype 860, median 565), 410–630 μm wide (holotype 620, median 460), broadest near mesothorax and metathorax. Body outline oval, nearly circular in smaller individuals (< 600 μm long), becoming elongate-oval in larger individuals. Derm membranous throughout at maturity except for pygidium. Antennae simple, each with one long seta. Distance between antennae 40–100 μm. Without disc pores associated with anterior or posterior spiracles. ***Lobes*.** Pygidium with 2 pairs of lobes; L1 well developed, separated by space about one-fifth width of L1, lobes slightly wider than long, inner margins near parallel, with 1 medial and 1 lateral notch, rounded apically; L2 forming sclerotized point, about one-quarter to one-third size of L1, with 1 lateral notch; L3 absent, indicated at most by small, lightly sclerotized projection of pygidial margin. ***Plates*.** All plates simple; with or without fine plates in slight space between L1; with 2 pointed plates in first space; plates absent in second space; five simple microduct-bearing plates present laterad of L3, nearly as long as L1. ***Ducts*.** Dorsal macroducts of 1-barred type, slender, with orifices narrower in diameter than ventral microducts, restricted primarily to margin with one submarginal duct anterior to seta marking segment VI; 1 between L1, with 3–4 marginal ducts in first space, 2 marginal ducts in second space; with few short macroducts occurring on submarginal areas of pre-pygidial segments. Ventral microducts slightly wider in diameter than dorsal macroducts and present in small submarginal groups on pre-pygidial abdominal segments and segment V. ***Paraphyses*.**L1 each with a paraphysis-like basal sclerosis toward medial margin, slightly smaller than lobe; in first space, 1 clavate paraphysis arising from lateral angle of L1, 1 arising from mesal angle of L2, posterior-most paraphysis slightly longer than L1; 2 smaller clavate paraphyses arising from mesal margin of L3. ***Anal opening*** oval, 8–13 μm in length, 4–6 μm in width, positioned 20–23 μm from base of L1, located within posterior third of pygidium. ***Perivulvar pores*** absent.

#### Remarks.

This species is placed in the genus *Clavaspis* MacGillivray on the basis of the robust clavate paraphyses, small anal opening, and basal sclerosis of L1, resembling that of *Clavaspisulmi* (Johnson). The paraphyses are not as elaborately developed as those of most *Clavaspis* species, but they are more developed than some species that have recently been recognized as members of *Clavaspis* on the basis of molecular phylogenetics – *C.perseae* (Davidson) and *C.patagonensis* Schneider, Claps, Wei, Normark & Normark (Normark et al. 2014; Schneider et al. 2020). *Clavaspisvirolae* is similar to *Clavaspisulmi*, but differs in having L2 present, plates fewer, dorsal macroducts fewer, medial paraphysis of first space less developed, and ventral macroduct orifices larger than those of dorsal macroducts. *Clavaspisvirolae* also resembles species of *Hemiberlesia* Cockerell, especially *H.ignobilis* Ferris and *H.ocellata* Takagi & Yamamoto, but differs in having a smaller anal opening and fewer plates. It further differs from *H.ignobilis* in having L2 present and ventral macroduct orifices larger than those of dorsal macroducts, and from *H.ocellata* in having 2 pairs of conspicuous paraphyses present, L3 absent, and notching of L1 and L2 less deep. Yet another genus that *C.virolae* resembles is *Diaspidiotus* Berlese: the axes of L1 and L2 seem to converge slightly, causing the species to key out as *Quadraspidiotus* MacGillivray, now a synonym of *Diaspidiotus*, in Ferris’s (1942) key. But this is not as good a fit, as *Diaspidiotus* species lack basal scleroses of L1. It is also biogeographically less plausible, as *Diaspidiotus* is overwhelmingly a temperate Holarctic group. There exist Neotropical species assigned to *Diaspidiotus*, but these may be misplaced. The only such species reported from Panama, *D.crescentiae* Ferris, has a large anal opening and basal scleroses of L1, and is best regarded as *Hemiberlesiacrescentiae* (Ferris), new combination.

#### Host plant.

*Virolamultiflora* (Standl.) A.C.Sm. (family Myristicaceae)

#### Etymology.

The specific epithet is the Latin genitive of the host plant genus, *Virola*.

#### Distribution.

Panama (Colón).

**Figure 2.**
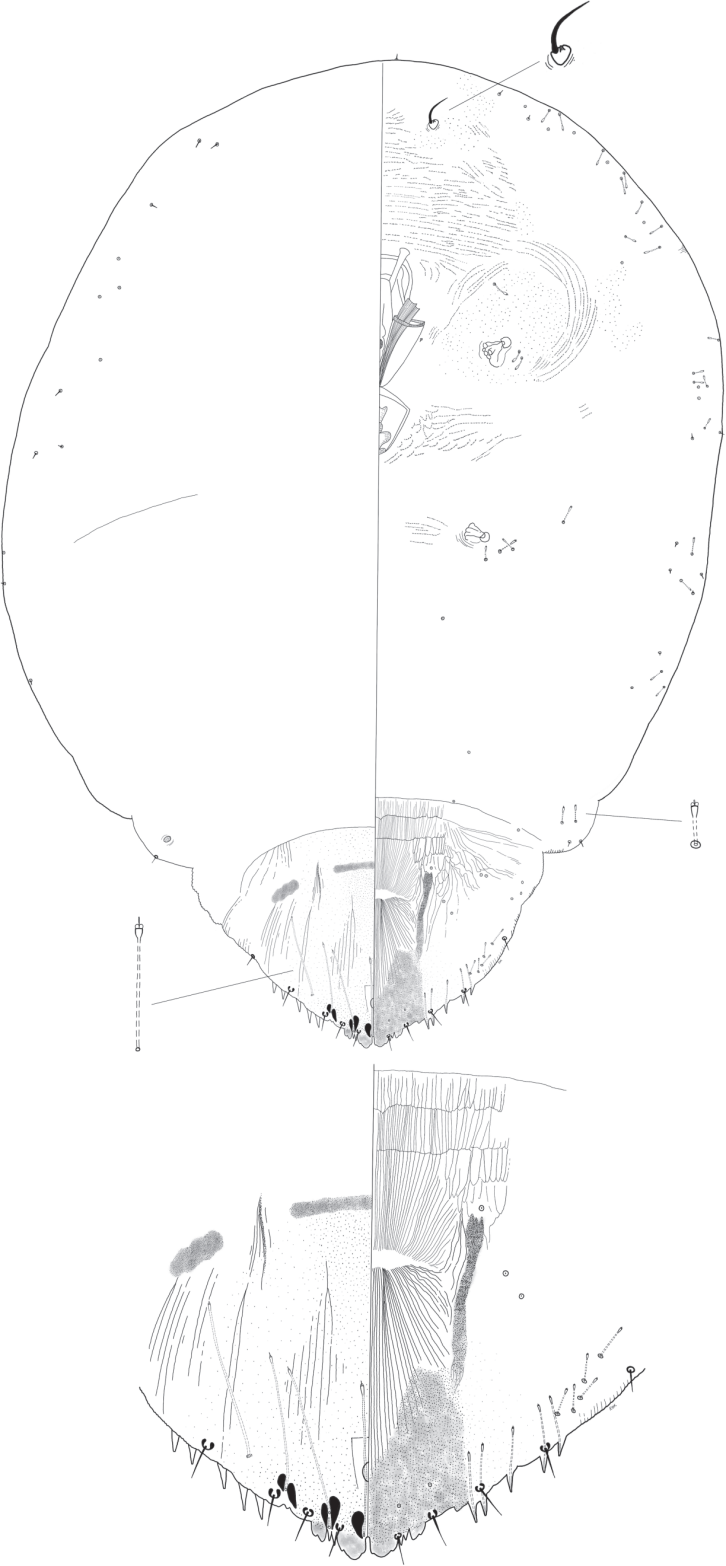
*Clavaspisvirolae* Wei, Schneider, Normark & Normark sp. nov. Adult female, full body view, illustrated from the holotype (D6676B); expanded view of pygidium, illustrated from the holotype (D6676B).

### Davidsonaspis tovomitae

Taxon classificationAnimaliaHemipteraDiaspididae

Wei, Schneider, Normark & Normark sp. nov.

AF12E3F1-DF68-5462-846C-5FF31919BEA1

http://zoobank.org/EE712529-F0A9-4EAA-AB50-20F29BB27DF7

Figure 3

#### Material examined.

***Holotype*:** Panama • 1 adult female; Parque Nacional San Lorenzo Canopy Crane, Colón; 9.2802°N, 79.9754°W; 12.vi.2012, GE Morse & BB Normark leg.; on *Tovomitalongifolia*; MIUP (D3919A). ***Paratype***: Panama • 1 adult female; Parque Nacional San Lorenzo Canopy Crane, Colón; 9.2802°N, 79.9754°W; 15.i.2015; DA Peterson, GE Morse, H Shapiro, S Trujillo leg.; on *Tovomitalongifolia*; UMEC (D6433A).

#### Description

**(*N* = 2). Adult female** not pupillarial. Appearance in life not recorded. Slide-mounted adult female 870–1060 μm long, 670–790 μm wide, broadest at mesothorax. Body outline broadly obovate. Antennae simple, each with one conspicuous long seta. Distance between antennae 160 μm. Without any disc pores associated with anterior or posterior spiracles. ***Lobes***. Pygidium with 3 pairs of lobes extending out from posterior margin, well sclerotized. L1 large, apically convergent, each lobe oval in shape, with minute notch near midpoint of outer margin; L2 distinctly smaller than L1, with 1 or 2 small notches on outer margin; L3 similar in size and shape to L2, with 1 or 2 notches on outer margin and 0 or 1 notch on inner margin. ***Plates*.** Without plates between L1; with 2 plates between L1 and L2, flabellate, apically fringed, each slightly longer than L1, much longer than L2; three between L2 and L3, flabellate, apically fringed, each plate longer than L3; three anterior to L3, branched and elaborately fringed on apical and lateral margins, much longer than L3, each with internal microduct. ***Ducts*.** Dorsal macroducts of 1-barred type, dorsal submarginal macroducts about same size as marginal macroducts, long (120–140 μm) and narrowly ribbonlike, with minute orifices, few, only 10–15 on each side of pygidium. Also, with faux duct orifice on dorsum immediately anteriad of L1 – circular structure slightly larger than duct orifices, but without duct. Pre-pygidial dorsal macroducts few, shorter than those on pygidium, confined to margin and submargin, absent on segments III and IV, two present on each side of segments II, I, metathorax, and mesothorax. Ventral microducts shorter and thinner than dorsal macroducts, with a few present on submargin of each segment from abdominal segment V to prothorax. ***Paraphyses*.** Three pairs of paraphyses present on each side of pygidium, variable, with lateral member of each pair often minute or absent. Medial pair of paraphyses anteriad of L1, medial member of pair arising from near inner angle of L1, extending nearly to anus and terminating in rounded knob, lateral member of pair minute, forming part of sclerotized rim of faux duct orifice; pair of paraphyses between L1 and L2 also with medial paraphysis much larger than lateral paraphysis; pair between L2 and L3 usually about equal to each other in length, lateral member of pair sometimes obsolete. ***Anal opening*** nearly circular, maximum diameter 8 μm, located 23–25 μm (about 3 times diameter) from base of L1. ***Perivulvar pores*** absent.

#### DNA sequences.

DNA sequences from 3 loci of the holotype of *Davidsonaspistovomitae* have been published: the large ribosomal subunit (28S; GenBank accession number KY219920), elongation factor 1-alpha (EF-1α; KY221745), and carbamoylphosphate synthetase (CAD; MH916177). The small ribosomal subunit (16S) sequences of the primary bacterial endosymbiont, *Uzinuradiaspidicola*, of the holotype has also been published (KY220578).

#### Informal synonyms.

The holotype of *D.tovomitae* has appeared in published phylogenetic trees, where it was labeled “Davidsonaspis ud3919” (Schneider et al. 2018) or “Davidsonaspis undescr” (Normark et al. 2019).

#### Remarks.

The only other known species in this genus is *Davidsonaspisaguacatae* (Evans, Watson, and Miller), found on avocados in Mexico. *D.aguacatae* had originally been assigned to *Abgrallaspis* Balachowsky (Evans et al. 2009), but was later reassigned to a new genus *Davidsonaspis* Normark (Normark et al. 2014). The new species can be distinguished from *D.aguacatae* in having a series of 3 plates anterior to L3, each as broad as L3 and elaborately fringed on apical and lateral margins; in *D.aguacatae*, plates anterior to L3 are narrower than L3 and only slightly fringed. *D.tovomitae* otherwise closely resembles *D.aguacatae*, and the two species form a clade in published molecular phylogenetic trees (Schneider et al. 2018; Normark et al. 2019). The structure we refer to as a faux duct orifice anteriad of L1 is illustrated by Evans et al. (2009) but not mentioned in their description. In one of their 2 illustrations of the pygidium of *D.aguacatae* the structure is shown with a central dot, as if it were the circular base of a seta, but in *D.tovomitae* no seta is present there.

#### Host plant.

*Tovomitalongifolia* (Rich.) Hochr. (family Clusiaceae)

#### Etymology.

The specific epithet is the Latin genitive of the host plant genus, *Tovomita*.

#### Distribution.

Panama (Colón).

**Figure 3.**
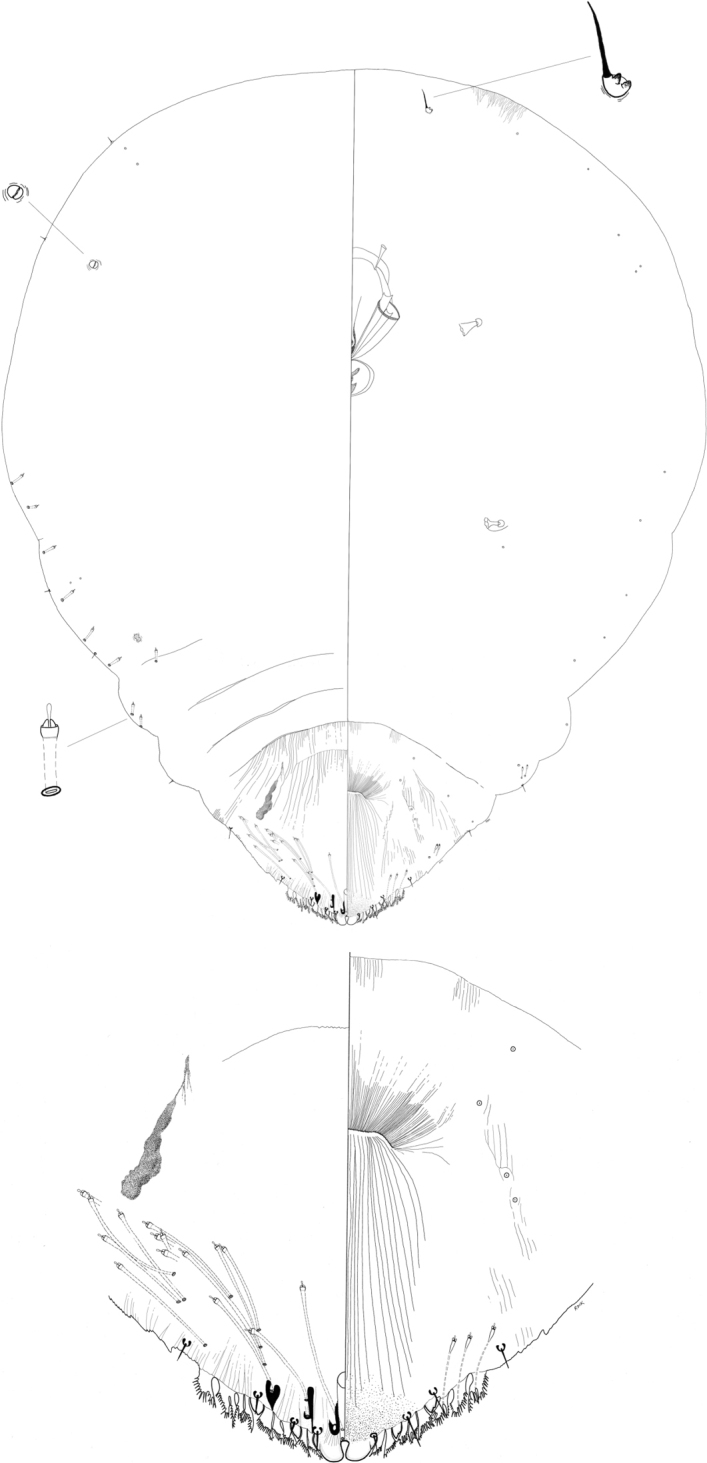
*Davidsonaspistovomitae* Wei, Schneider, Normark & Normark sp. nov. Adult female, full body view, illustrated from the holotype (D3919A); expanded view of pygidium, illustrated from the holotype (D3919A).

### Rungaspis neotropicalis

Taxon classificationAnimaliaHemipteraDiaspididae

Wei, Schneider, Normark & Normark sp. nov.

5DFB5CD3-8322-5043-9D6C-CA4FF1DF05D6

http://zoobank.org/02D416A8-3589-4AC4-877A-3F3A88E7C59B

Figures 4 , 5

#### Material examined.

***Holotype*:** Panama • 1 adult female; Parque Nacional San Lorenzo Canopy Crane, Colón; 9.2802°N, 79.9754°W; 20.vi.2012; GE Morse & BB Normark leg.; on *Marilalaxiflora* Rusby; MIUP (D4168I). ***Paratypes***: • 4 adult females; same data as holotype; USNM (D3953K, D4168B, D6550C, D6552B); • 5 adult females; same data as holotype; UMEC (D3953J, D3953P, D3995B, D4168E, D6703C).

#### Description.

**Adult female** (*N* = 10) in some cases pupillarial, enclosed within sclerotized cuticle of 2^nd^ instar; some individuals non-pupillarial. Appearance in life not recorded. Slide-mounted adult female 350–610 μm long (holotype 540 μm, median 540 μm), 280–500 μm wide (holotype 410 μm, median 420 μm), broadest at mesothorax. Body outline broadly oval, with slight indentation between prothorax and mesothorax. Derm membranous throughout at maturity in pupllarial individuals; cephalothorax and pygidium becoming sclerotized at maturity in some non-pupillarial individuals. Antennae simple, each with one long seta. Distance between antennae 51–73 μm. Eye a submarginal dorsal tubercle on prothorax. Without disc pores associated with anterior or posterior spiracles. Venter of mesothorax with about 6 transverse, irregular rows of sclerotized spicules in submedial area, posterolaterad of mouthparts. ***Lobes*.** Pygidium with 1 or 2 pairs of lobes; L1 well developed, subquadrate, with parallel inner margins separated by exceedingly narrow space, lobes slightly longer than wide, rounded apically, with 1 large notch near apex on lateral margin and 0–1 notch near apex on medial margin; L1 each with well-developed basal sclerosis, slightly narrower and longer than lobe; L2, when fully developed, forming a small, sclerotized projection, about one-third length of L1 and much narrower, without notches; L2 often absent or represented by a membranous projection or low, sclerotized point; L3 absent. ***Plates*.** With 2 narrow, elongate plates in first space, slightly fringed, with a few tines, and 1 or 2 simple plates laterad of position of L2; no other plates present. ***Ducts*.** Dorsal macroducts of 1-barred type, slender, much broader than ventral microducts, few in number, restricted to margin of pygidium; with 1–3 (usually 2) ducts in first space, 0–2 (usually 1) immediately laterad of L2, and 0–1 (usually 0) laterad of seta marking segment VI, making a total of only 1–4 ducts (usually 4) on each side of pygidium. Ventral microducts exceedingly narrow, present along pygidial margin and scattered in submedial areas of other segments. ***Paraphyses*** absent. ***Anal opening*** subcircular, 8–11 μm in length and width, positioned 17–37 μm from base of L1, located within posterior half of pygidium. ***Perivulvar pores*** absent.

**Figure 4.**
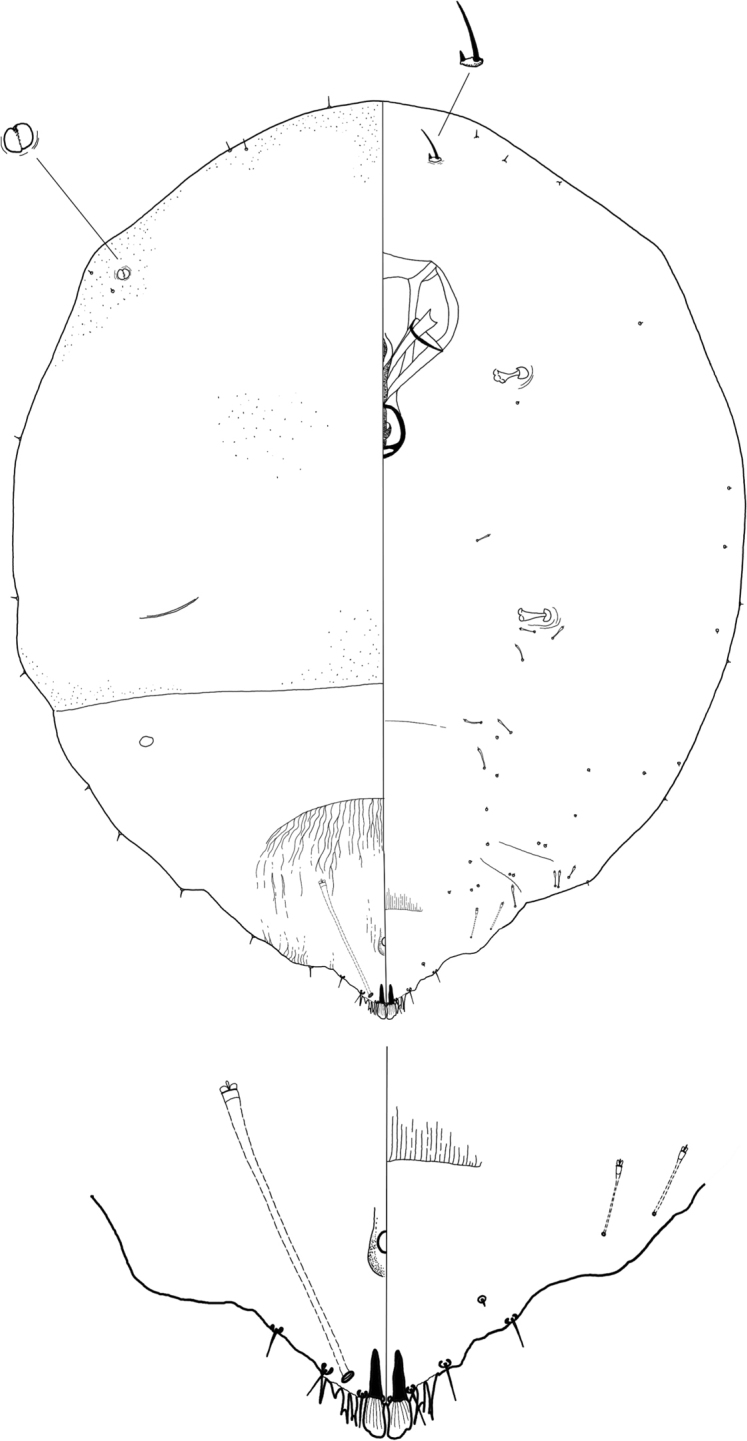
*Rungaspisneotropicalis* Wei, Schneider, Normark & Normark sp. nov. Adult female, full body view, illustrated from the holotype (D4168I); expanded view of pygidium, illustrated from the holotype (D4168I).

**Second-instar female** (*N* = 8) Appearance in life not recorded. Slide-mounted second-instar female 340–620 μm long (median 460 μm), 270–400 μm wide (median 340 μm), broadest at mesothorax. Body outline oval. Antennae simple, each with one long seta. Distance between antennae 54–96 μm. Without disc pores associated with anterior or posterior spiracles. ***Lobes*.** Pygidium with 3 pairs of well-developed lobes; L1 subquadrate, with parallel inner margins separated by exceedingly narrow space, lobes slightly longer than wide, rounded apically, with 1 large notch near apex on lateral margin and 0–1 notch near apex on medial margin; L1 each with well-developed basal sclerosis, slightly narrower and longer than lobe; L2 nearly as long as L1 but much narrower, rounded at apex, without notches or with slight notch on lateral margin; L3 subtriangular, slightly narrower and shorter than L2, without notches. ***Plates*.** Without plates between L1. With 2 narrow plates in first space, 2 broader plates in second space, and a series of 5 or 6 plates laterad of L3. All plates similar in length to adjacent lobes and fringed at apex, with plates anterior to L3 becoming progressively lower and less fringed anteriorly. Plates of the first and second spaces subtended by conspicuous ducts, about a third as wide as dorsal macroducts and nearly as long, much wider and longer than ventral microducts. ***Ducts*.** Dorsal macroducts of 1-barred type, broad, all submarginal; with 2 ducts in a short row arising from first space, 2 in the second space, and 1 laterad of L3, making a total of 5 on each side of pygidium. Ventral microducts exceedingly narrow, short, present along pygidial margin and scattered in submedial areas of other segments. ***Paraphyses*** absent. ***Anal opening*** oval to subcircular, 8–14 μm in length, 7–8 μm in width, positioned 23–40 μm from base of L1, located within posterior half of pygidium.

#### DNA sequences.

Several DNA sequences of *Rungaspisneotropicalis* have been published, including fragments of 3 gene regions: the large ribosomal subunit (28S; D3953H, D3953J, D3953R, D3953V, D4168B, D4168E,D4168I, D4168J, D4249H, D4249L; Genbank accession numbers MT677181–MTT677184, MTT677266–MTT677296, MT677294), elongation factor 1-alpha (EF-1α; D3953J, D3953V, D3953W, D3995B; D4168A, D4168B, D4168E, D4168J, D4249H, D4249L; KY221749, MH915953, MH915954, MT64783, MT642022, MT642025–MT642029, MT642031, MT642032), and cytochrome oxidase I and II (COI–II; D3953H, D3953J, D3953R, D3953V, D3995B, D4168A, D4168B, D4168E, D4168I, D4168J, D4249G, D4249H, D4249L; KY221137, MH916549, MT676875–MT676878, MT676946–MT676950, MT676971, MT676972, MT676974).

#### Informal synonyms.

Specimens of *R.neotropicalis* have appeared in published analyses and phylogenetic trees, where they were labeled “UG3995 ud3995” (Schneider et al. 2018; Normark et al. 2019), “UG3953 ud3953” (Schneider et al. 2018), or “Rungaspis ud3995” (Peterson et al. 2020).

#### Remarks.

This is an unusual species both in its life history, showing intraspecific variation in the pupillarial habit, and in its biogeography, having affinities to African species. Some slide-mounted specimens are unequivocally pupillarial, having well-developed 1^st^ instars inside of adult females that are themselves inside of 2^nd^-instar cuticles. More often than not, these adult females are flipped inside their puparia, with their head at the posterior end of the puparium. Other specimens are apparently non-pupillarial, and some of these have a sclerotized cephalothorax, a feature not seen, to our knowledge, in adult females of any pupillarial species. We had originally intended to describe the pupillarial and non-pupillarial forms as two different species, but the three sequenced gene regions show no differences between them and there are no consistent morphological differences either; therefore, we consider them to comprise a single species that includes both pupillarial and non-pupillarial developmental phenotypes. The second instar has a more completely developed secretory system than the adult, with more ducts, plates, and lobes – a pattern typical of pupillarial species and opposite to what is typical of non-pupillarial species. This may imply that this species is derived from a pupillarial ancestor and that the non-pupillarial form represents a secondary loss of the pupillarial habit.

Molecular phylogenetic studies have shown that *R.neotropicalis* has affinities with African species. Probably the best analysis is a recent study of Aspidiotini (Schneider et al. 2018), which shows *R.neotropicalis* nested within a clade of African *Aspidiotus* species (*A.fularum* Balachowsky, *A.elaeidis* Marchal, and an undescribed species from Uganda), with *R.neotropicalis* sister to *A.fularum*. *R.neotropicalis* was also included in a broader study of Diaspididae (Normark et al. 2019), where it appears in a clade that consists mostly of African species (*A.elaeidis*, *Selenaspiduskamerunicus* Lindinger, *S.articulatus* Morgan, *Dynaspidiotusrhodesiensis* (Hall), and *Entaspidiotuslounsburyi* (Marlatt)) but that also includes one other New World species (*Rugaspidiotusarizonicus* (Cockerell)). It is possible that *R.neotropicalis* is an African species that is invasive in the Neotropics, similar to *Selenaspidusarticulatus*, which is the single most abundant diaspidid species at the site where *R.neotropicalis* was collected (Peterson et al. 2020). But if this species is from Africa, it does not seem to have ever been found there. Based on the available evidence we regard it as a native Neotropical species, perhaps one resulting from an ancient trans-Atlantic dispersal event.

**Figure 5.**
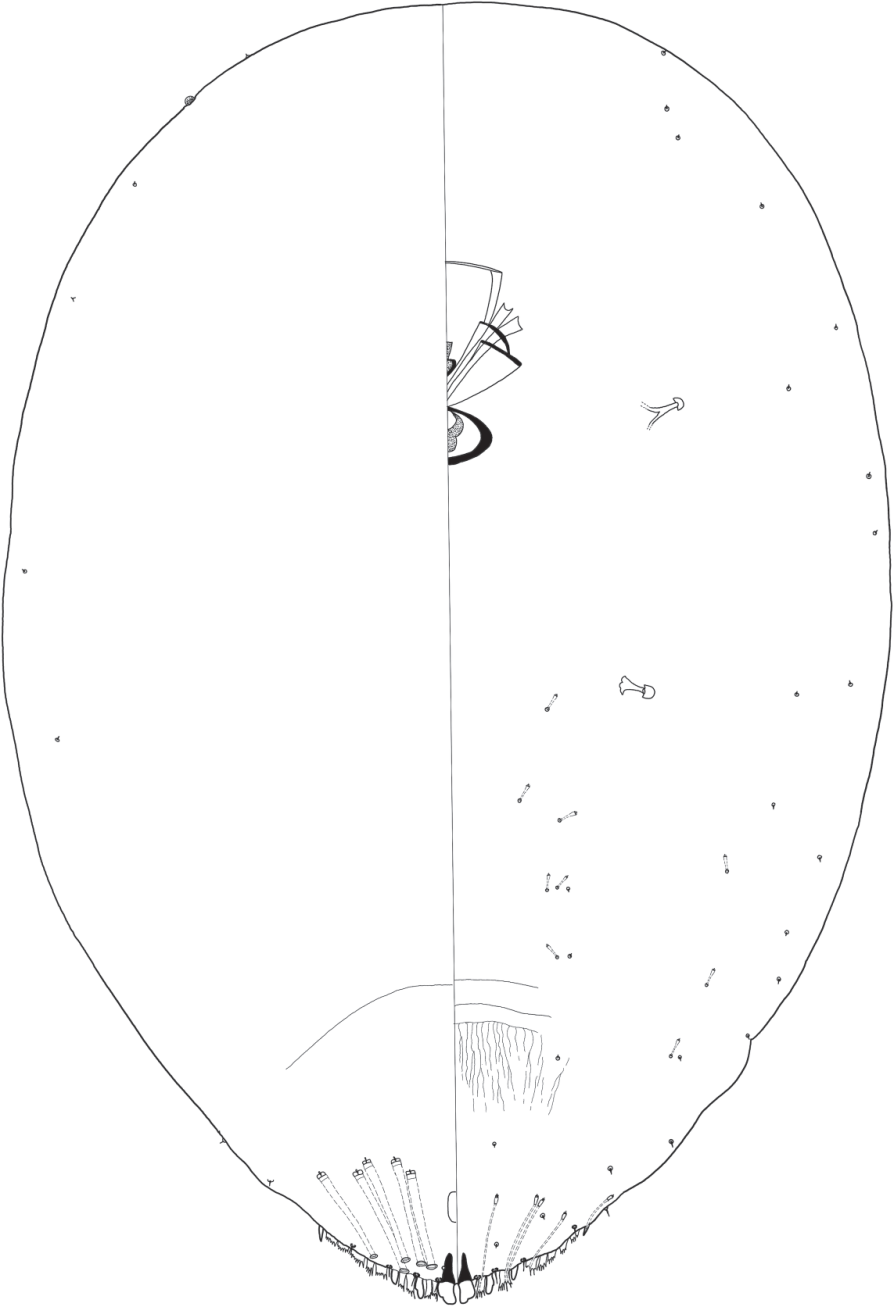
*Rungaspisneotropicalis* Wei, Schneider, Normark & Normark sp. nov. Second-instar female, full body view.

We tentatively place this species in the genus *Rungaspis* Balachowsky. *Rungaspis* presently comprises four species distributed in Africa and the southwestern Palearctic. *Rungaspisneotropicalis* resembles the other species of *Rungaspis* in having large basal scleroses of L1, reduced L2 and L3, cephalothoracic sclerotization at maturity (in non-pupillarial specimens), dorsal ducts with sclerotized orifices, and simplified plates located only in the first and second interlobular spaces. African *Rungaspis* species differ from *R.neotropicalis* in having conical plates without fringes (vs. slightly fringed) and numerous narrow dorsal ducts (vs. few broad ducts). We considered three other possible placements for the species. One was the genus *Aspidiotus* Bouché. *Rungaspisneotropicalis* resembles *Aspidiotus* species in having basal scleroses of L1 and fringed plates, and molecular evidence indicates that its closest known relative is an African species of *Aspidiotus*. But we concluded that *R.neotropicalis* shares a greater number of characters with *Rungaspis*. Furthermore, *Aspidiotus* is radically non-monophyletic, and the mostly African clade to which *R.neotropicalis* belongs should probably be recognized as a distinct genus anyway (Schneider et al. 2018). Another possible placement we considered was the genus *Helaspis* McKenzie. *Helaspis* is a New World genus that “appears to suggest *Aspidiotus* more strongly than any known genus” (McKenzie 1963). With *R.neotropicalis* it shares basal scleroses of L1 and a sclerotized cephalothorax. But *Helaspis* has other extraordinary features – conical plates and bilobed L3 – that seem to indicate an affinity with the tribe Gymnaspidini rather than Aspidiotini (Normark et al. 2019). *Rungaspisneotropicalis* lacks these characters and is clearly a member of Aspidiotini. We also considered erecting a new genus for *R.neotropicalis* – this is the course taken by many diaspidid systematists faced with such an unusual species – but we concluded that that was not appropriate in this case given the evidence for affinity with *Rungaspis*.

Morphologically, *R.neotropicalis* also closely resembles *Aspidiotusrhusae* (Brain), a pupillarial species known from South Africa. The two species share a similar overall body shape, L1 with basal scleroses, absence of L3, absence of perivulvar pores, and presence of just a few slightly fringed plates and just a few broad, one-barred dorsal ducts near the pygidial margin. Characters that distinguish *R.neotropicalis* from *A.rhusae* are as follows (character of *A.rhusae* given in parentheses): L2 much narrower than L1 or absent (L2 nearly as broad as L1); space between L1 exceedingly narrow, without plates (space between L1 with pair of apically fringed plates); 4 or fewer dorsal ducts present on each side of pygidium (5 or more ducts present); 1–3 microducts present near each posterior spiracle (cluster of 5 or more ducts in this position); transverse rows of minute spicules present on mesothorax posterolaterad of mouthparts (absent); body margin slightly indented between prothorax and mesothorax (entire); eye a submarginal dorsal tubercle (eye marginal). The Neotropical species that *R.neotropicalis* most closely resembles is *Aspidiellarigida* Ferris. The two species both have L1 with basal scleroses and closely approximated medial margins, other lobes reduced or absent, cephalothorax becoming sclerotized at full maturity, and perivulvar pores absent. Characters that distinguish *Rungaspisneotropicalis* from *Aspidiellarigida* are as follows (character of *A.rigida* given in parentheses): plates present (absent); dorsal ducts of pygidium broad, much broader than ventral microducts, confined to margin and submargin (narrow, similar to ventral microducts, widely scattered); anus in posterior half of pygidium (anterior half).

Our study of Neotropical and African species that resemble *Rungaspisneotropicalis* has further led us to conclude that *Aspidiellarigida* belongs in the genus *Rungaspis*, and we transfer it accordingly: *Rungaspisrigida* (Ferris) comb. nov. Ferris (1941) remarked, “It is with much doubt that this species is here referred to the genus *Aspidiella*. In its pygidial characters it resembles the type genus closely enough except for the entire absence of plates and the absence of the perivulvar pores... In the heavy sclerotization of the entire body it is peculiar and distinctive.” In each of these characters it resembles *Rungaspis* species more than *Aspidiella* species. Ferris further expressed puzzlement that an Oriental and Australian genus such as *Aspidiella* would include a species that was apparently native to the Neotropics. A biogeographic connection between the Neotropics and Afrotropics is better documented (by *Rungaspisneotropicalis* and in groups such as *Diaspis* Bouché) and less of a surprise.

#### Host plant.

*Marilalaxiflora* Rusby (family Calophyllaceae)

#### Etymology.

The specific epithet is a Latin adjective; here it alludes to this species’ unusual biogeography as a Neotropical member of a mostly African clade.

#### Distribution.

Panama (Colón).

### Selenaspidopsis browni

Taxon classificationAnimaliaHemipteraDiaspididae

Nakahara, 1984: 936. New country record

C1CCEC04-2D3E-5AAE-81BF-36378DBB8C3E

#### Material examined.

Panama • 1 adult female; Parque Metropolitano Canopy Crane; 8.9944°N, 79.5431°W; 22.i.2015; DA Peterson, GE Morse, H Shapiro, S Trujillo leg.; on *Antirheatrichantha*; MIUP (D6765D); • 1 adult female; same data as previous; UMEC (D6765G).

#### Host plant.

*Antirheatrichantha* (Griseb.) Hemsl. (Rubiaceae)

#### Distribution.

Panama (Parque Metropolitano).

### Key to species of Aspidiotini from Panama based on adult females

The key incorporates some modified excerpts drawn from Ferris (1942), Deitz and Davidson (1986), Smith-Pardo et al. (2012) and Normark et al. (2014). The key excludes *Hemiberlesiapaucitatis* (McKenzie) due to insufficient information.

**Table:** 

1	With deep thoracic constriction between prothorax and mesothorax or mesothorax and metathorax	**2**
–	Without deep thoracic constriction on thorax	**3**
2	With deep thoracic constriction between mesothorax and metathorax; L3 spur-shaped, distinctly different from L2; perivulvar pores in 2 groups on pygidium (*Selenaspidus*)	***Selenaspidusarticulatus* (Morgan)**
–	With deep thoracic constriction between prothorax and mesothorax; L3 similar in shape to L2; perivulvar pores in 4 groups on pygidium (*Selenaspidopsis*)	***Selenaspidopsisbrowni* Nakahara**
3	Paraphyses absent on pygidium	**4**
–	Paraphyses present on pygidium	**11**
4	Perivulvar pores absent	**5**
–	Perivulvar pores present	**8**
5	With L3 definitely developed and easily distinguishable from pygidial margin	**6**
–	L3 lacking, at most represented by crenulations of pygidial margin (*Rungaspis*)	**7**
6	L4 absent, plates beyond L3 simple (*Chortinaspis*)	***Chortinaspissubchortina* (Laing)**
–	L4 present, plates beyond L3 apically fringed (*Nigridiaspis*)	***Nigridiaspisarmigera* Ferris**
7	Dorsal ducts present in submedian areas of pygidium; entire body strongly sclerotized at maturity	***Rungaspisrigida* (Ferris) comb. nov.**
–	Dorsal ducts absent from submedian areas of pygidium; cephalothorax slightly sclerotized at maturity or body membranous	***Rungaspisneotropicalis* sp. nov.**
8	L3 well developed and similar in shape to L2; pygidial macroduct orifices distinctly wider than ventral microducts; any plates present anterior to L3 deeply fringed (*Aspidiotus*)	**9**
–	L3 poorly developed and dissimilar in shape to L2; pygidial macroduct orifices not much wider than ventral microducts; any plates present anterior to L3 simple or minimally fringed (*Aspidiella*)	**10**
9	Pre-pygidial marginal macroducts absent; with total of 15–29 dorsal macroducts on each side of body	***Aspidiotusdestructor* Signoret**
–	Pre-pygidial marginal macroducts present; with total of 22–38 dorsal macroducts on each side of body	***Aspidiotusexcisus* Green**
10	Plates present anterior to L3; plates between L1 equal to or slightly longer than lobes, clearly visible; L3 represented by unsclerotized point	***Aspidiellahartii* (Cockerell)**
–	Plates absent anterior to L3; plates between L1 about same length as lobes, somewhat obscured by lobes; L3 represented by swelling of margin only slightly larger than protrusions along remainder of pygidium	***Aspidiellasacchari* (Cockerell)**
11	Prosoma of mature female reniform; with 3 long fleshy plates laterad of L3; paraphyses short and indistinct; abdominal segments I–III with submarginal groups of macroducts (*Aonidiella*)	***Aonidiellaorientalis* (Newstead)**
–	Prosoma of mature female elongate, round, oval or turbinate, not reniform; combination of plates laterad of L3, paraphyses, and pre-pygidial macroducts not as described above	**12**
12	Body elongate and more or less parallel-sided, 3× or 4× as long as wide (*Pseudischnaspis*)	**13**
–	Body round, turbinate, or oval, less than 3× as long as wide	**14**
13	Body long and quite slender, cephalic margin almost straight; apical angle of pygidium more than 90 degrees; perivulvar pores in 5 groups	***Pseudischnaspisacephala* Ferris**
–	Body elongate but broad, cephalic margin broadly rounded; apical angle of pygidium less than 90 degrees; perivulvar pores in 4 groups	***Pseudischnaspisbowreyi* (Cockerell)**
14	Most paraphyses shorter than or similar in length to L1, generally less than 2× its length; sometimes with 1 pair of paraphyses longer than L1 arising from first interlobular space and terminating in an abruptly swollen knob; never bearing paraphyses anterior to position of L3	**15**
–	Paraphyses typically longer than L1, often exceeding 2× its length; with more than 1 pair of paraphyses exceeding length of L1, which either remain thin throughout or gradually expand apically; sometimes bearing paraphyses anterior to position of L3	**28**
15	Anal opening relatively large, distance between posterior edge of opening and base of L1 usually not more than 2× diameter of anal opening; plates usually with fringed apices (except 1 species bearing simple plates) (*Hemiberlesia*)	**16**
–	Anal opening small, distance between posterior edge of opening and base of L1 usually greater than 2× diameter of anal opening; plates simple or minimally fringed (*Clavaspis*)	**24**
16	Perivulvar pores absent	**17**
–	Perivulvar pores present	**21**
17	Having the following combination of characters, plates in the first and second interlobular spaces all simple, L2 and L3 entirely absent	***Hemiberlesiacrescentiae* (Ferris) comb. nov.**
–	Without this combination, at least some fringed plates present in first and second interlobular spaces, L2 and L3 at least indicated by a hyaline plate-like lobe	**18**
18	Plates anterior to position of L3 absent, simple, or fringed, but without protruding central microduct	**19**
–	Plates anterior to position of L3 trifurcate, consisting of central protruding marginal microduct and 2 lateral processes that may be simple or fringed	**20**
19	L1 with short, broad basal sclerosis, projecting anteriorly; L2 and L3 sclerotized and distinct from plates	***Hemiberlesiamusae* Takagi & Yamamoto**
–	L1 without broad basal sclerosis (with paraphysis-like sclerotization at base of medial or lateral margin in some specimens); L2 and L3 hyaline and plate-like	***Hemiberlesiaignobilis* Ferris**
20	L2 and L3 hyaline; L1 subsemicircular, divergent; each plate between L1 and L2 with 1 associated microduct	***Hemiberlesiaandradae* Okusu & Normark**
–	L2 and L3 sclerotized; L1 with 1 lateral notch, closely appressed and parallel; each plate between L1 and L2 with 2 or 3 associated microducts	***Hemiberlesiadiffinis* (Newstead)**
21	L2 definitely developed, sclerotized, distinctly dissimilar to a pygidial plate	**22**
–	L2 represented by unsclerotized point or lobe, similar in appearance to a pygidial plate	**23**
22	L3 represented by sclerotized point without notches; plates beyond L3 variously fringed; eyes represented by distinct spurs	***Hemiberlesiacyanophylli* (Signoret)**
–	L3 pointed but with at least 1 lateral notch and 0-1 medial notches; plates beyond L3 minimally fringed; eyes indistinct, not represented by spurs	***Hemiberlesiamendax* McKenzie**
23	Plates deeply fringed, definitely exceeding L1 in length, all similar in size and shape including plates beyond L3; L3 sclerotized	***Hemiberlesiapalmae* (Cockerell)**
–	Plates shallowly fringed, only slightly exceeding L1 in length, varying in size and shape, plates beyond L3 simple; L3 unsclerotized	***Hemiberlesialataniae* (Signoret)**
24	Paraphyses arising from lateral angle of L1 typically elongate, slender and terminating in a sclerotized swollen knob	**25**
–	Paraphyses arising from lateral angle of L1 clavate but not terminating in a sclerotized swollen knob	***Clavaspisvirolae* sp. nov.**
25	Perivulvar pores present, at least 1 pore per side	**26**
–	Perivulvar pores entirely absent	**27**
26	With 2 plates between L1 and L2; submarginal groups of microducts form semicircle around head, thorax and pre-pygidial abdominal segments; with at least 2 large macroducts on mesothorax	***Clavaspisselvatica* sp. nov.**
–	With 1 plate between L1 and L2; submarginal groups of microducts not organized in obvious semicircular ring; with only 1 large macroduct on mesothorax	***Clavaspiscoursetiae* Marlatt**
27	With L1 alone being well developed; plates fringed and equal in length to L1	***Clavaspisherculeana* (Cockerell & Hadden)**
–	With 4 pairs of well-developed lobes; plates simple and much shorter than L1	***Clavaspisdentata* Ferris**
28	Paraphyses arising only from basal angles of lobes or position of obsolete lobes, never from within interlobular spaces; paraphyses in first interlobular space typically about 2× longer than those in second interlobular space (although nearly identical in length for 1 species); perivulvar pores absent (*Palinaspis*)	**29**
–	With at least 1 paraphysis arising from an interlobular space; paraphyses in first and second interlobular spaces not following this pattern; perivulvar pores present or absent	**31**
29	Plates reduced to short membranous lobes, rounded apically	***Palinaspislobulata* Ferris**
–	Plates present or absent, if present, elongate	**30**
30	Plates entirely lacking; with 1 notch on each side of each lobule L1; L2 entirely lacking or at most represented by very slight irregularity of margin	***Palinaspissordidata* Ferris**
–	Plates well-developed; with 1 notch on outer margin of each lobule of L1; L2 represented only by low, slightly sclerotized swelling of margin	***Palinaspisbarbata* Ferris**
31	Having combination of 3 pairs of pygidial lobes, 1 paraphysis arising from first interlobular space, and lacking paraphyses beyond L3	***Davidsonaspistovomitae* sp. nov.**
–	Without above combination; paired paraphyses in first space arise from outer angles of lobes, with 3–4 well-developed lobes and with or without paraphyses present beyond L3	**32**
32	Pygidial margin anterior to L4 not heavily sclerotized, without series of short paraphyses; plates anterior to L3 conspicuous, branched, fringed or clubbed, usually exceeding length of lobes; anus usually located closer to posterior margin than to vulva (*Chrysomphalus*)	**33**
–	Pygidial margin anterior to L4 heavily sclerotized, often with series of short paraphyses; plates anterior to L3 not as long and conspicuous, may be simple, branched, fringed or spine-like, usually not exceeding length of lobes; anus usually in center of pygidium or closer to vulva than posterior margin	**34**
33	First 2 plates anterior to L3 with clavate apices; pre-pygidial segments lacking dorsal cluster of 4 or more ducts	***Chrysomphalusdictyospermi* Morgan**
–	First 2 plates anterior to L3 with fringed apices; abdominal segment II with dorsal cluster of 5 or more ducts along lateral margin	***Chrysomphalusaonidum* (Linnaeus)**
34	Pygidium long and narrow, sharply tapering to acute apical point, lateral margins slightly concave; with 3 pairs of pygidial lobes, L4 reduced to point or absent; margin anterior to L4 heavily sclerotized; most paraphyses between L1 to L4 elongate (***Acutaspis***)	**35**
–	Pygidium short and broad, not tapering apically to acute point, lateral margins convex; usually with 4 or 5 pairs of pygidial lobes, L4 usually well developed; margin anterior to L4 lightly to heavily sclerotized; paraphyses between L1 to L4 variable in shape	**38**
35	Lateral thoracic margin produced into distinct point or rounded umbo near posterior spiracles	**36**
–	Lateral thoracic margin without such point or umbo, at most with small, sclerotized spot in this position	**37**
36	Lateral thoracic margins produced into very pronounced umbos; sclerotization of derm developed in sharply defined marginal zone extending from umbos, along sides, and across pygidium	***Acutaspisumbonifera* (Newstead)**
–	Umbos quite small, sclerotization forming similar pattern as above, but rather weakly developed	***Acutaspisperseae*** (**Comstock**)
37	1 very long paraphysis arising from outer angle of L3, 1 or 2 small paraphyses arising from base of L4; pre-pygidial dorsal ducts present; derm membranous except for pygidium	***Acutaspisreniformis* (Cockerell)**
–	1 long paraphysis arising from outer angle of L3, small paraphyses absent from base of L4; pre-pygidial dorsal ducts absent; derm strongly sclerotized at full maturity	***Acutaspisalbopicta* (Cockerell)**
38	Pygidium with longest paraphyses arising from lateral angles of lobes; large V-shaped reticulate sclerotized area on abdominal segment VI always present (*Crenulaspidiotus*)	**39**
–	Pygidium with longest paraphyses arising from interlobular spaces; without large V-shaped reticulate sclerotized area on abdominal segment VI	**40**
39	With 5 pairs of lobes; with 2 plates anterior to L4; ventral microducts present between L3 and L4; paraphysis formula normally 1-1-1	***Crenulaspidiotusmaurellae* (Laing)**
–	With 4 pairs of lobes, without plates anterior to L4; ventral microducts absent between L3 and L4; paraphysis formula normally 1-2-1	***Crenulaspidiotussinuatus* (Ferris)**
40	Anterior head margin of mature female forming distinctly sclerotized prominence, differentiated from lateral margin, resembling a “cap” (*Mycetaspis*)	**41**
–	Anterior head margin of mature female not distinctly sclerotized or differentiated from lateral margin, not resembling a “cap” (*Melanaspis*)	**44**
41	Perivulvar pores present in 5 small groups; cephalic area very heavily sclerotized and bearing series of conspicuous setae	***Mycetaspissphaerioides* (Cockerell)**
–	Perivulvar pores absent; cephalic area without series of conspicuous setae	**42**
42	L1 each with elongate, tapering basal sclerosis with base about as wide as base of L1	***Mycetaspispersonata* (Comstock)**
–	L1 with basal sclerosis narrow and arising from mesal angle, with base less than half as wide as base of L1	**43**
43	With sclerotized spur on head; longest paraphysis in third interlobular space arising from center of interlobular space, posterior to L4	***Mycetaspisapicata* (Newstead)**
–	Without sclerotized spur on head; longest paraphysis in third interlobular space arising near mesal angle of L4	***Mycetaspisdefectopalus* Ferris**
44	Perivulvar pores present	**45**
–	Perivulvar pores absent	**47**
45	Perivulvar pores present in 5 small groups; pygidium moderately acute at apex, lateral margins almost straight; first interlobular space with moderately long, apically swollen paraphysis followed by small process from mesal angle of L2	***Melanaspisnigropunctata* (Cockerell)**
–	Perivulvar pores present in 4 small groups; pygidium with lateral margins convergent; first interlobular space with quite long paraphysis followed by very small process from mesal angle of L2	**46**
46	L1 with very small basal process, without long paraphysis between them; without long paraphysis arising from outer angle of L3, with short and broad paraphysis arising from inner angle of L4	***Melanaspisponderosa* Ferris**
–	With long paraphysis between L1; with long paraphysis arising from outer angle of L3; without paraphysis arising from inner angle of L4	***Melanaspisbondari* Lepage & Giannotti**
47	Pygidial lobes each with dorsal seta sunk to at least 0.25× length in distinct socket	***Melanaspissulcata* Ferris**
–	Pygidial lobes with dorsal setae not in distinct sunken sockets	**48**
48	With median band of dermal reticulations, squamations or transverse striations (median squamations may be indistinct)	**49**
–	Without median band of dermal reticulations, squamations or transverse striations (median striations, if present, longitudinal)	**50**
49	Third interlobular space with longest paraphysis about equal in length to longest paraphysis in first and second interlobular spaces; venter without long microducts	***Melanaspiscoccolobae* Ferris**
–	Third interlobular space with longest paraphysis much shorter than longest paraphysis in first space or second interlobular spaces; venter with long microducts along pygidial margin anteriorly and in 2 irregular submarginal rows posterior to ventral pygidial scar	***Melanaspissquamea* Ferris**
50	With only 1 paraphysis in third interlobular space; with long microducts in 3 distinct longitudinal bands on each side of vulva on venter	***Melanaspislongula* Ferris**
–	With various numbers of paraphyses in third interlobular space; with long microducts not arranged in distinct longitudinal bands on each side of vulva, or with 2 or fewer such bands	**51**
51	Usually with reduced paraphyses in third interlobular space; orifices of macroducts large and conspicuous in dorsal sclerotized areas	**52**
–	Usually with 1 or more well-developed paraphyses in third interlobular space; orifices of macroducts smaller and less conspicuous in dorsal sclerotized areas	**54**
52	With 3 paraphyses in second interlobular space; ventral seta in middle of or anterior to base of lobe on each L1	***Melanaspissmilacis* (Comstock)**
–	With 2 paraphyses in second interlobular space; ventral seta laterad of base of lobe on each L1	**53**
53	Orifices of macroducts absent on lateral margin of dorsal sclerotized area 2; paraphyses in third interlobular space nearly equal in length	***Melanaspisodontoglossi* (Cockerell)**
–	Orifices of macroducts present on lateral margin of dorsal sclerotized area 2; paraphysis arising from outer angle of L3 slightly longer than others in third interlobular space	***Melanaspiseglandulosa* (Lindinger) (in part)**
54	Anal opening between or only slightly anterior to apices of paraphyses; without definite paraphyses beyond L4	***Melanaspistenebricosa* (Comstock)**
–	Anal opening decidedly anterior to apices of all paraphyses; with paraphyses beyond L4	**55**
55	Pygidial margin with 2 or 3 conspicuous, spur-like processes anterior to L4	***Melanaspisleivasi* (Costa Lima)**
–	Pygidial margin without spur-like processes anterior to L4	**56**
56	Anal opening located within posterior third of pygidium from base of median lobe; with 5 paraphyses in third interlobular space	***Melanaspistenax* (McKenzie)**
–	Anal opening located near center of pygidium; with 3–4 paraphyses in third interlobular space	**57**
57	Without macroduct orifices on membranous area in third interlobular space; all macroduct orifices with equal diameters	***Melanaspislatipyga* Ferris**
–	With macroduct orifices on membranous area in third interlobular space; macroduct orifices in third interlobular space usually smaller in diameter than any macroduct orifices located more mesally (on abdominal segments VI–VIII)	***Melanaspiseglandulosa* (Lindinger) (in part)**

Additional online resources aiding in the identification of Aspidiotini are provided by Schneider et al. (2019) and Dooley (2006).

## Supplementary Material

XML Treatment for Clavaspis selvatica

XML Treatment for Clavaspis virolae

XML Treatment for Davidsonaspis tovomitae

XML Treatment for Rungaspis neotropicalis

XML Treatment for Selenaspidopsis browni
